# Collaboration across continents to produce e-learning for palliative care education in Sub Saharan Africa

**DOI:** 10.3332/ecancer.2014.ed36

**Published:** 2014-05-27

**Authors:** FM Rawlinson, E Luyirika

**Affiliations:** 1 Consultant in Palliative Medicine, University of Wales College of Medicine, Velindre Hospital, Cardiff CF14 2TL, Wales, UK; 2 Executive Director, African Palliative Care Association, P. O. Box 72518, Plot 95, Dr Gibbons Road, Makindye, Kampala, Uganda

Cancer in Africa remains a challenge with survival rates affected by late detection, delay in seeking diagnosis, unavailability of technical support for diagnosis, lack of resource for treatment and the availability of palliative care [1], [2]. The number of new cancer cases in Africa is predicted to increase to 1.28 million with 970,000 deaths each year by 2030 [3]. Competing health priorities for the continent and the high costs associated with cancer awareness, diagnosis and treatment mean that the issues with diagnosis, availability, affordability and accessibility of treatment are likely to continue. There is therefore an urgent need to increase the availability of palliative care provision.

Palliative care in Africa has been established since the late 1970’s with the first hospice being Island Hospice in Zimbabwe. The impact of palliative care on patients’ quality of life and survival is now well documented [4]. The Cape Town Declaration of 2002 asserted pain relief as a human right and the need for palliative care to be incorporated into national health care strategies [5]. The African Palliative Care Association (APCA), formed in 2004, is the leading organisation for Africa on advocacy, policy development and providing support for services across the continent. Recent milestones include the African Union’s Common Position on Controlled Substances and Access to Pain Management Drugs and the Declaration on Non-communicable Diseases (NCDs) which include palliative care [6]. and the development of a consensus statement for palliative care integration into health systems in Africa ‘Palliative care for Africa’. This followed the first African ministers of Health session in Johannesburg in September 2013 [7]. Despite the major developments of the last 40 years, expansion and development needs continue [8], [9].

Education remains vital to the effectiveness, recruitment, retention and sustainability of all palliative care teams [10]. However, delivering education across Africa involves a number of resource challenges: availability of educators, the impact on organisations of time away for learners for face to face sessions, travelling distances, logistics and cost [11–13].

Using technology to deliver education is not a new concept; increasing internet availability and access to mobile phone technology provide the opportunity to deliver education on a far greater scale [13–16]. A project, funded by the International Atomic Energy Agency (IAEA), part of a cancer management portfolio, was undertaken to develop open access learning in palliative care for cancer in sub Saharan Africa, in conjunction with the Virtual University of Cancer Control (VUCCnet). The aim was to create palliative care education content which was flexible to learners’ needs and time commitment.

Using education material from the Cardiff University palliative care ‘short course’ for GP’s as its basis, further material was sought. The aim was to work in collaboration with VUCCnet focal points across Africa and APCA, ensuring that this new education initiative would enhance material already in place [11–13], minimise duplication and deliver a programme that was accessible and relevant to the intended participants. Discussions around participant groups, content, delivery, certification and accreditation, practical experience and ongoing educational research took place in July, informing the subsequent shape of the project.

Creating the material involved filming interviews and teaching sessions with colleagues in UK and Africa and consultations with actor patients. The footage has been edited into 20 modules, each with fact slides and interactive quizzes. An introduction section sets out the aims, objectives and reflective practice prompts and includes a graded knowledge test to complete before opening the educational material. A prompt to collect outcome measures, either individually or as part of a service, using the APCA POS (Palliative care Outcome Scale) tool follows [17], [18]. Competing health priorities in Africa have led to the increasing importance of collecting outcome measures to demonstrate the effectiveness of palliative care; [19] the African POS has been validated specifically for this purpose. African research to underpin African service development and delivery remains a key guiding principle [9], [17]. Each module concludes with key points, reflective practice review, and an opportunity to repeat the graded knowledge test which, on satisfactory completion, prompts a certificate of achievement for that module. Finally, an evaluation form and resource slides with an additional prompt to seek some face to face experience with the learners’ local hospice team conclude the module.

There were some technical, practical and ethical challenges during the process, such as developing a ‘consent for filming’ form which could be understood by someone who may not have an understanding of the internet and never accessed such a technology. Other challenges included the handling of sensitive material on camera: patients’ distress around their situation as they outlined their experiences of having cancer, taking morphine for pain, the perceptions from their friends and family to both cancer and opioids and the acutely painful and poignant conversations with parents of a dying child in the last moments of life. The role of a palliative care consultant as an interviewer was useful to ensure that appropriate responses and support were given, although there were moments of internal conflict at times to continue to film an interview through the distress. The narrative of palliative care involves powerful language and meaning so appropriate debriefing and preparation also informed the effective placing of the cameras and supported the production team. The material was afterwards sensitively edited into the modules.

Checking content for relevance and appropriateness to the destination took place during the APCA/HPCA conference in September 2013. Twenty-nine testers reviewed all twenty modules and gave valuable feedback which informed subsequent interviews and the final editing. Formal peer reviewing from two reviewers per module, prior to publication, resulted in some final changes, mostly in relation to the African context. The majority of editing work following peer review was for the modules on cancer management, psychosocial and spiritual care.

The impact of any education on patient care and patient experience needs to be evaluated [20], [21] and for these modules, it is forming part of a longitudinal project. Baseline information on completion, and further evaluation at 3 months and 6 months is being sought. This enables content to be continually reviewed and updated as well as providing data for educational research.

The final product is a collection of 20 modules, free to access online which cover aspects of symptom control in palliative care, cancer and its management, end of life care, ethics, communication skills and resource management. Successful completion of graded questions on each module achieves a certificate of completion for the learner. For participants where online access is still intermittent, allowing for the fact that the impact of the audiovisual content is lessened, a paper version with transcripts of each film clip has been developed and is available on registration.

Delivering education on line in isolation will be partially effective but participants are encouraged to seek some face to face training and experience with their local palliative care /hospice teams if possible to directly apply their knowledge and build up links and resources [11–13]. In the future, a more formal link with local organisations as they develop is anticipated, as is using this model for fact delivery and self directed learning for other global developments. Translation to French, Portuguese and local languages is under discussion.

In summary, using developing technology to develop education enables creativity and opportunity. Creating educational material for a target audience or setting needs close collaboration with all potential stakeholders and a deep understanding of the context within which services are delivered in order to achieve maximal effect. It is possible to combine resources globally to produce meaningful and useful material. Appropriate and responsible use of resources would suggest that such material does not duplicate what is already available, but complements that material already present.

Ultimately, the final point of any education programme is the patient, and any education resource must have improved patient care at its heart.

## Online resources

Modules available at: www.ecancer.org/education

APCA: www.africanpalliativecare.org

Cardiff University palliative care education: http://courses.cardiff.ac.uk/postgraduate/course/detail/p233.html
